# Effects of Pregnancy on Oral Health: A Narrative Review

**DOI:** 10.7759/cureus.94929

**Published:** 2025-10-19

**Authors:** Anjani Varsha, Arnesh Garg, Rishabh Thakur

**Affiliations:** 1 Department of Oral Medicine and Radiology, Sri Ramachandra Institute of Higher Education and Research, Chennai, IND

**Keywords:** clinical implications, hormonal changes, oral health, pregnancy, preventive measures

## Abstract

Pregnancy induces significant physiological and hormonal changes that can affect oral health. These changes can worsen existing oral conditions or lead to the development of new conditions such as pregnancy gingivitis, periodontal diseases, and dental caries. This review explores the various factors responsible for these conditions, such as changes in immunological response, oral environment, increased levels of steroid hormones, and nutritional deficits. Periodontal diseases that are left untreated during pregnancy are also seen to be associated with adverse maternal and fetal outcomes. On the other hand, dental caries is further exacerbated due to salivary changes in the oral cavity of pregnant women. Hence, a comprehensive management of oral health during pregnancy should focus on prevention, patient education, and safe management of existing conditions. Dental treatment should be provided following the recommended guidelines for the different stages of pregnancy, with the second trimester being the safest for most procedures. A strong collaboration between dental professionals and prenatal care providers is essential for ensuring optimal maternal and fetal health. However, the presence of socioeconomic challenges, such as a lack of awareness and restricted access to dental treatment, underscores the need for developing targeted interventions and innovative solutions for delivering oral health education and preventive care to pregnant women.

## Introduction and background

Oral health is a critical component of overall health, particularly during pregnancy, marked by significant physiological and hormonal changes. These changes can exacerbate oral health issues or lead to new conditions, impacting maternal and fetal health. Pregnancy induces pronounced elevation of estrogen and progesterone levels, which markedly influence oral tissues by increasing gingival vascular permeability and compromising immune responsiveness, thereby heightening susceptibility to inflammation, even in the presence of minimal plaque [1]. These hormonal changes foster alterations in the oral microbiome and immune environment, promoting gingival hyperplasia, bleeding, and swelling commonly identified as pregnancy gingivitis [1,2]. In more severe instances, localized overgrowths such as pyogenic granulomas may develop, particularly during the second and third trimesters, along with other gum-related diseases such as chronic periodontitis [1,2]. Furthermore, untreated periodontal disease during pregnancy has been associated with adverse maternal and fetal outcomes, such as preterm birth and low birth weight, through mechanisms involving systemic dissemination of oral pathogens and inflammatory mediators [3,4]. Understanding these interactions underscores the importance of integrating oral health screenings and preventive strategies into prenatal care to safeguard both maternal and fetal well-being, which is vital for healthcare providers to offer comprehensive prenatal care. This review aims to summarize recent findings and synthesize current research on the effects of pregnancy on oral health, emphasizing the need for integrated healthcare approaches to improve outcomes.

## Review

Methodology

The article was designed to be a narrative review, and so ethical approval was not sought. The articles were selected from various sources such as PubMed, Scopus, MedScience, etc., compiling data from 1960 to 2025 after screening their abstracts based on their relevance, contents, and updates. Articles written in English only were included. The keywords used while searching for relevant articles included "pregnancy," "oral health," and "hormonal changes."

Hormonal changes and oral health

Pregnancy is characterized by increased levels of estrogen and progesterone, which play a pivotal role in altering oral health. These hormones, attaching to receptors in the gingival, salivary, and periodontal tissues, enhance vascular permeability, connective tissue remodeling, and immunological modulation, hence elevating susceptibility to gingival inflammation even with less plaque. Estrogen stimulates the proliferation of blood vessels, leading to increased gingival vascularity and sensitivity [5]. Progesterone enhances the inflammatory response to plaque, contributing to gingival inflammation [6]. Studies have shown that hormonal changes can also affect the oral microbiome, potentially increasing susceptibility to periodontal disease [7]. Additionally, hormonal shifts during pregnancy can impact salivary composition and flow rate, further predisposing pregnant women to oral health issues [8]. The reduction in saliva flow and changes in its composition can decrease the oral cavity's natural defense mechanisms, making it more susceptible to infections and caries. In postmenopausal women, a decline in estrogen reduces saliva flow and buffering capacity, impairing mucosal integrity and elevating the risk of periodontal disease progression [9]. These hormone-mediated changes underline the critical need for vigilant oral hygiene and targeted preventive care during hormonally active phases.

Common oral health issues during pregnancy

Pregnancy Gingivitis

This condition affects a significant proportion of pregnant women, with studies indicating prevalence rates as high as 60%-75% when including people from various races [10]. Several studies have been done on the occurrence of pregnancy gingivitis. Research has shown that it can occur as early as the first trimester, although it is predominantly noticed and most common in the second trimester and peaks during the third trimester [11,12]. Characterized by inflammation, swelling, and bleeding of the gums, pregnancy gingivitis can progress to more severe periodontal disease if not managed adequately. The condition is primarily caused by increased blood flow to the gums and changes in the body’s response to oral bacteria [13]. Nutritional factors such as iron deficiency anemia and vitamin C deficiency can lead to spontaneous bleeding and other obstetric problems during pregnancy, which reach their peak in the third trimester [14]. Effective management includes regular dental visits, improved oral hygiene practices, and possibly the use of antimicrobial mouth rinses. 

Periodontitis

Approximately 30 to 40% of expectant mothers develop periodontitis as a result of untreated gingivitis [15]. Periodontitis is a chronic inflammatory disease affecting both the connective tissue and the surrounding alveolar bone [16]. It is characterized by a major shift in the oral microbiome composition, largely due to the presence of key anaerobic pathogens [17]. 

While the primary etiological agent responsible for periodontal tissue damage is the presence of bacteria within dental plaque biofilms, there are also additional factors that can lead to oral changes and subsequently harm the periodontium. One such factor is the hormonal fluctuations during pregnancy. Various hormones, including sex steroids, can affect the cellular components of periodontal tissues and may disrupt the processes involved in the development of periodontitis [18]. Estrogen and progesterone, in particular, affect periodontal tissues by altering connective tissue cell turnover, impairing vascular response, and increasing gingival vasculature permeability. This creates pathways for periodontal pathogens to enter the circulation and potentially infect fetal-placental units [19]. Common periodontal pathogens found in the fetal-placenta unit include *Aggregatibacter actinomycetemcomitans*, *Prevotella intermedia*, *Campylobacter rectus*, *Tannerella forsythia*, *Treponema denticola*, and *Fusobacterium nucleatum* [20,21]. These bacteria were classified into distinct complexes or clusters, each identified by a specific color: purple, yellow, green, orange, and red [22]. Among these, the orange cluster plays a crucial role in biofilm maturation and facilitates colonization of red-complex organisms, including *Porphyromonas gingivalis*, *Bacteroides forsythus*, and *Treponema denticola* [23]. In addition to this, periodontal issues can cause many systemic conditions, such as adverse pregnancy outcomes and nutritional disorders.

A proposed hypothesis suggests a relationship between elevated maternal hormones and an increase in periodontal pathogens through direct and indirect pathways. Pathogens like *P. intermedia* and *P. gingivalis* have been illustrated to directly exploit these hormones to synthesize vitamin K, which is crucial for their maturation and proliferation [23]. Indirectly, higher hormone levels alter gingival parameters by increasing probing depth and the secretion of gingival crevicular fluid, affecting gingival keratinization and reducing immune response [24]. A 2010 study reported significant increases in bacterial counts during the second and third trimesters of pregnancy [25]. Additionally, higher salivary progesterone levels were found to be significantly associated with *P. gingivalis* counts [26].

Tooth Mobility

Tooth mobility, an indicator of periodontal disease, arises from mineral alterations in the lamina dura and disruptions in the attachments of the periodontal ligament. A lack of vitamin C has been identified to exacerbate. The treatment protocol involves elimination of gingival irritants, vitamin C supplementation, and targeted treatment delivery, which typically leads to a reversal of tooth mobility [27].

Dental Caries

Pregnant women are at a higher risk for dental caries, which are primarily caused by *Streptococcus mutans*, as well as other non-mutans *streptococci* like *Actinomyces* and *Lactobacillus*. This risk increases due to a combination of physiological and behavioral changes, such as increased oral acidity, sweet food cravings, insufficient oral hygiene, and postponed treatments, which collectively promote the formation and accumulation of biofilm [28-31]. 

Contrary to previous assumptions that teeth experience calcium loss during pregnancy [32], studies on the chemical composition of human dentine have revealed no significant difference in the mineral content between the teeth of pregnant and non-pregnant women [33]. However, pregnancy does alter saliva composition by decreasing its levels of calcium and phosphate [34,35]. These changes, particularly in late pregnancy and lactation, increase the risk of caries development due to factors such as increased demineralization (lowered buffer effect and pH), decreased remineralization, and higher levels of mutans streptococci in saliva. Morning sickness, a common occurrence in pregnancy, may further increase the risk of dental erosion [8].

Due to the multifactorial causes of caries, no single element plays a significant role in their development. It appears that pregnancy primarily influences the environmental aspects of teeth, such as alterations in salivary gland function and composition, rather than affecting the teeth directly. Changes in saliva during pregnancy may contribute to caries formation in mothers who already have other risk factors [8].

Pregnancy Tumours

These are benign growths that occur in the oral cavity during pregnancy. They generally are without any pain or numbness, but the patient may have bleeding and mild discomfort [36]. These tumors, also referred to as pyogenic granulomas, usually resolve postpartum but may require removal if they interfere with oral function or hygiene. Their development is linked to increased levels of progesterone and can be exacerbated by local irritants like plaque. Identical lesions with the same histological structure occur along with florid gingivitis and periodontitis that may complicate pregnancy, which are referred to as pregnancy epulis. The frequency of these increases during the end of pregnancy and then shrinks after delivery, which may imply that they are directly proportional to the estrogen levels [1].

Systemic implications of oral health

Periodontitis in pregnant women has been associated with various adverse pregnancy outcomes (APOs), including preterm birth, low birthweight, pre-eclampsia, and gestational diabetes mellitus (GDM). Preterm birth accounts for 75% of perinatal mortality and over 50% of long-term morbidity [37,38]. Approximately 15 million preterm births occur annually, with incidence rates varying globally: 5-9% in Europe, 12% in the United States, and up to 15% in developing countries [39]. Low birth weight affects over 20 million babies annually worldwide, with a global incidence rate of 14.6%. Notably, 91% of low-birth-weight cases occur in low- and middle-income countries, primarily in Southern Asia and sub-Saharan Africa [40]. Preeclampsia affects 5-8% of pregnant women and is recognized by the World Health Organization as the third leading cause of maternal death [41,42]. A recent meta-analysis reported that women with periodontitis have twice the odds of developing GDM compared to those without periodontitis, after adjusting for confounding factors [43]. However, these findings underscore the need for more robust prospective studies to confirm and elucidate the relationship between periodontal health and adverse pregnancy outcomes [44].

**Figure 1:**
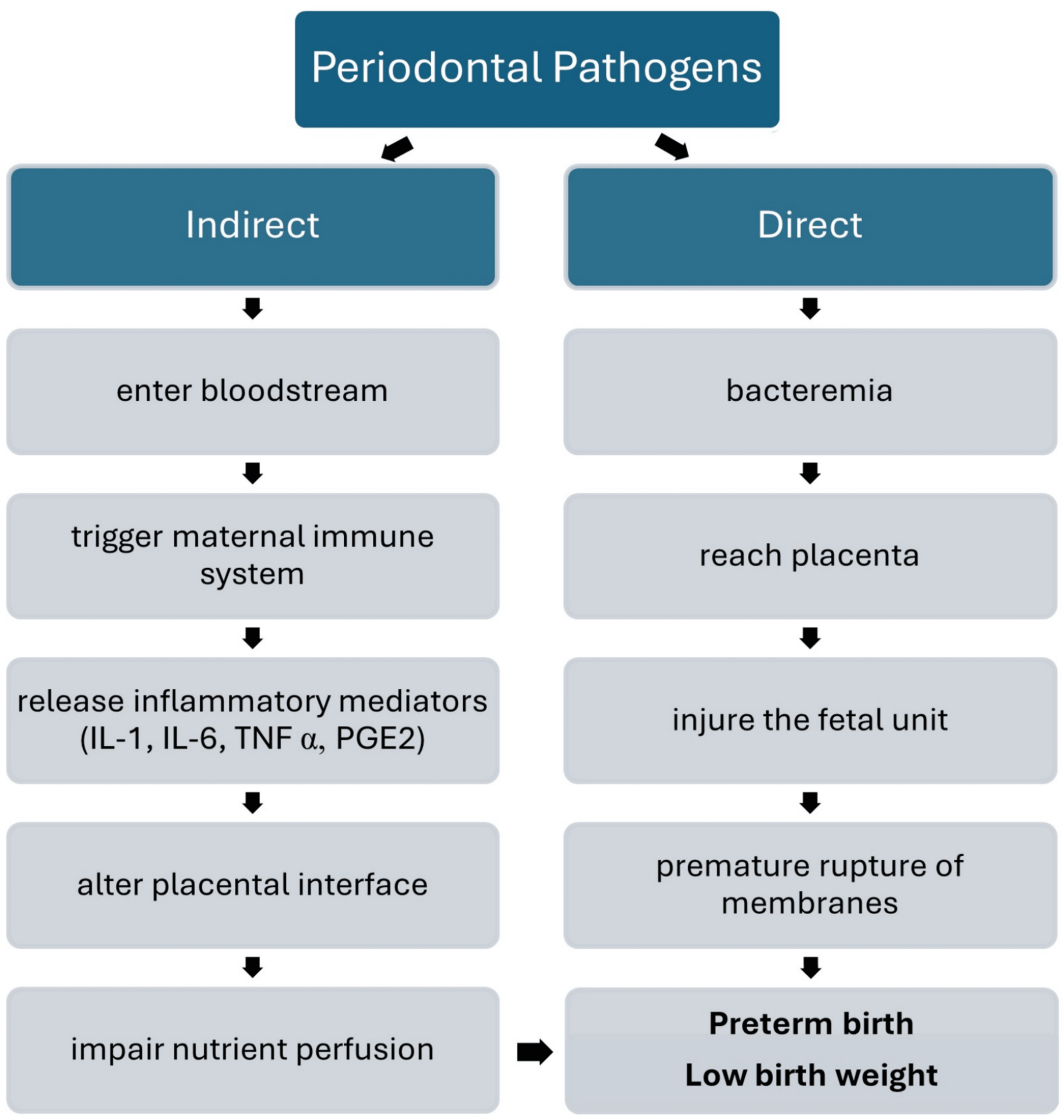
Association between periodontal disease and adverse pregnancy outcomes Created by the authors of this study IL-1: interleukin-1, IL-6: interleukin-6, TNF α: tumor necrosis factor alpha, PGE2: prostaglandin E2

Two possible explanations for this relationship are the direct migration of periodontal pathogens to the fetoplacental unit and the indirect action of inflammatory mediators like prostaglandin E2 (PGE2), interleukin-1 (IL-1), IL-6, IL-8, and tumor necrosis factor-alpha (TNF-α), which can all cause a systemic reaction (Figure 1) [45]. The liver increases and spreads fibrinogen and C-reactive protein in response to this trigger, which both exacerbate inflammation [45]. Moreu et al. from Spain demonstrated a favorable correlation between elevated maternal probing depth and the incidence of preterm, low-birth-weight infants. They failed to link maternal periodontal disease to preterm delivery, but they did consider it a risk factor for preterm low birth weight [46]. According to reports, gestational diabetes mellitus may raise the risk of developing diabetes and obesity even after giving birth, as well as other unfavorable pregnancy outcomes such as gestational hypertension, miscarriage, and poor fetal growth and development. On evaluating parameters like probing depth and clinical attachment levels in both healthy individuals and those diagnosed with gestational diabetes mellitus, it has been discovered that the latter had worse periodontal conditions. Actually, periodontitis was detected in or has been reported to occur in 65% of mothers with gestational diabetes mellitus [47].

The link between periodontal disease and negative pregnancy outcomes is currently debated, possibly due to factors such as varying sample sizes, ethnicities, ages, lifestyles, and socioeconomic backgrounds, as well as differing definitions of periodontal disease and adverse pregnancy outcomes in research. To address these issues, future research should aim to increase sample sizes, control for confounding factors, and establish standardized diagnostic criteria for both periodontal disease and adverse pregnancy outcomes [48].

Oral health management during pregnancy

Dental care during pregnancy should focus on prevention and the safe management of existing conditions. Patient education on maintaining oral hygiene and recognizing symptoms of oral disease is also crucial. Safe practices during dental procedures, such as minimizing radiation exposure and using local anesthesia, should be followed [49]. Additionally, dental professionals should provide personalized advice on nutrition and oral hygiene tailored to the needs of pregnant women.

Nutritional Considerations

An important factor in preserving dental health is diet. It is recommended that pregnant women eat a well-balanced diet full of calcium, vitamin D, vitamin C, and phosphorus, all of which are vital for dental health. Another way to lower the risk of dental caries is to limit sugary foods and beverages. Studies have shown that folic acid helps to maintain periodontal health and reduce gingival inflammation [50]. Furthermore, adequate intake of omega-3 fatty acids has been associated with reduced inflammation and may benefit periodontal health during pregnancy.

Timeline and Recommendations for Dental Treatment

While early studies warned against dental work in the first trimester because of possible teratogenic risks during organogenesis and a peak in spontaneous abortions at the same time, the American College of Obstetricians and Gynecologists and the American Dental Association now state that full oral health care is safe and should be done throughout pregnancy. Preventive, diagnostic, and restorative procedures (such as local anesthesia and appropriately protected radiographs) should not be delayed, according to both organizations. This is due to the fact that postponing necessary care can exacerbate infections in expectant mothers and increase the likelihood of unfavorable birth outcomes. Routine and urgent dental work can still be done in every trimester as long as standard precautions are taken, but patients may still wish to postpone elective cosmetic procedures for their own comfort.

American College of Obstetricians and Gynecologists (ACOG) Guidelines

According to the American College of Obstetricians and Gynecologists, all pregnant patients should have a full dental check-up and clear advice on oral health at each stage of pregnancy. The organization emphasizes the need to keep up preventive habits, like brushing twice a day with fluoride toothpaste, flossing daily, and getting dietary advice and oral screening done during prenatal visits to reduce the risk of cavities. Patient education through maternal health programs on maintaining oral hygiene and recognizing symptoms of oral disease is also crucial. ACOG supports performing essential restorative and emergency procedures with local anesthetics like lidocaine, which are safe during pregnancy, without any trimester restrictions. When X-rays are necessary for diagnosis or treatment planning, they recommend using proper shielding for the abdomen and thyroid to limit fetal exposure. To enhance maternal comfort and safety during treatment, they suggest positioning the patient on her left side with slight head elevation to prevent supine hypotension [51].

American Diabetes Association (ADA) Guidelines

The American Dental Association also says that standard precautions make it safe to get cleanings, fillings, and extractions during all three trimesters. They allow the use of local anesthesia and X-ray imaging with the right shielding when it is clinically appropriate. They stress the importance of quickly treating any active oral problems to lower the risk of maternal infection and possible problems during pregnancy. Cosmetic procedures that aren't necessary, like teeth whitening or bonding, can wait until after the baby is born for the patient's comfort. However, routine and urgent dental care can be done at any time during pregnancy. The American Diabetes Association (ADA) also stresses how important it is to work with a patient's obstetric provider to find the best time for treatment and make sure that all aspects of prenatal health are covered [52].

When prescribing medications, healthcare providers must evaluate the associated risk using the Food and Drug Administration (FDA) classification, which categorizes drugs into five groups based on their scientific reliability and cost/benefit analysis. Local anesthetics are generally considered safe for use during pregnancy; however, their selection should be based on FDA guidelines and the specific treatment requirements [53]. To ensure comprehensive care and overall safety, dentists should maintain open communication and collaborate closely with the patient's physician throughout the course of treatment [54].

Psychological and Socioeconomic Factors

It is fundamental to remember that emotions and anxiety are accentuated during pregnancy, and this can intensify the fear and perception of pain in the dental chair [55]. Furthermore, socioeconomic status can impact access to dental care, with lower-income women facing more significant barriers. It is of utmost importance to consider these issues through public health initiatives and educate the public. Programs aimed at improving access to affordable dental care and increasing awareness about the importance of oral health during pregnancy can have a positive impact [56]. Community-based interventions and outreach programs can help bridge the gap in dental care access and education for underserved populations. Child Core Set and Adult Core Set are the 2025 Core Set of Children's Health Care Quality Measures for Medicaid and the Children's Health Insurance Program (CHIP), and the 2025 Core Set of Adult Health Care Quality Measures for Medicaid, respectively, which were released by the Centers for Medicare & Medicaid Services (CMS) in May 2024 [57].

Current research and future directions

Recent studies continue to explore the complex relationship between oral health and pregnancy outcomes. Future research should focus on teledentistry and longitudinal microbiome studies to better understand the causality and mechanisms involved, like hybrid effectiveness-implementation trials such as stepped-wedge and SMART/MRT designs [58]. Diagnostic-accuracy studies have also been done validating patient-captured intraoral images/video for common pregnancy-related conditions such as pregnancy gingivitis, erosive tooth wear, and mucosal lesions [59]. Additionally, developing targeted interventions to improve oral health in pregnant populations could have significant public health benefits. Research on the genetic and environmental factors contributing to oral health disparities among pregnant women is also needed [61]. Advances in digital health technologies and telemedicine may offer innovative solutions for delivering oral health education and preventive care to pregnant women, especially in remote areas.

## Conclusions

The influence of pregnancy on oral health is substantial, contributing to a spectrum of conditions that affect both maternal and fetal outcomes, thereby emphasizing the necessity of advancing evidence-based preventive strategies, early diagnostic protocols, and integrated clinical interventions. Hormonal changes during pregnancy can exacerbate conditions such as gingivitis, periodontal disease, and dental caries, which, if left untreated, may contribute to adverse pregnancy outcomes like preterm birth and low birth weight. Integrating dental care into routine prenatal services is not merely beneficial but imperative, ensuring early detection, prevention, and management of oral diseases; therefore, incorporating standardized oral health screening into every obstetric visit should be prioritized as a public health policy. Such an approach not only safeguards the mother’s oral and systemic health but also promotes healthier pregnancy outcomes. Ongoing research and public health initiatives remain critical to increasing awareness, reducing disparities, and improving access to comprehensive dental care for expectant mothers.
